# The influence of recipient SLCO1B1 rs2291075 polymorphism on tacrolimus dose–corrected trough concentration in the early period after liver transplantation

**DOI:** 10.1007/s00228-020-03058-w

**Published:** 2021-01-02

**Authors:** Yi Wu, Fang Fang, Zhaowen Wang, Peihao Wen, Junwei Fan

**Affiliations:** 1 Department of Hepatobiliary Pancreatic Surgery, Shanghai General Hospital, Shanghai Jiao Tong University School of Medicine, Shanghai, 200080 China; 2 Department of Nursing, Shanghai General Hospital, School of Medicine, Shanghai Jiao Tong University, Shanghai, 200080 China; 3 Department of Hepatobiliary and Pancreatic Surgery, The First Affiliated Hospital of Zhengzhou University, Zhengzhou, 450003 China; 4 Department of General Surgery, Shanghai General Hospital, Shanghai Jiao Tong University School of Medicine, Shanghai, 200080 China

**Keywords:** Live transplantation, Tacrolimus, CYP3A5, SLCO1B1, Polymorphism

## Abstract

**Purpose:**

To explore the relationship between rs2291075 polymorphism in SLCO1B1 gene, which encodes an influx transmembrane protein transporter, and tacrolimus dose–corrected trough concentration (C/D, ng ml^−1^ mg^−1^ kg^−1^) in the early period after liver transplantation.

**Methods:**

CYP3A5 rs776746 and SLCO1B1 rs2291075 polymorphisms of 210 liver transplantation patients and their corresponding donor livers were assessed by PCR amplification and DNA sequencing. The influence of gene polymorphisms on C/D values of tacrolimus was analyzed. The early postoperative period after liver transplantation was divided into the convalescence phase (1–14 days) and stationary phase (15–28 days) according to the change of liver function and tacrolimus C/D values.

**Results:**

The combined analysis of donor and recipient CYP3A5 rs776746 could distinguish the metabolic phenotype of tacrolimus into three groups: fast elimination (FE), intermediate elimination (IE), and slow elimination (SE), which was entitled the FIS classification system. Tacrolimus C/D ratios of recipient SLCO1B1 rs2291075 CT and TT carriers were very close and were significantly lower than those of recipient SLCO1B1 rs2291075 CC genotype carriers in convalescence phase (*p* = 0.0195) and in stationary phase (*p* = 0.0152). There were no statistically significant differences between tacrolimus C/D ratios of patients carried with SLCO1B1 rs2291075 CT, TT genotype donors, and those carried with SLCO1B1 rs2291075 CC genotype donors. A model consisting of tacrolimus daily dose, total bilirubin, FIS classification, and recipient SLCO1B1 rs2291075 could predict tacrolimus C/D ratios in the convalescence phase by multivariate analysis. However, recipient SLCO1B1 rs2291075 genotype failed to enter forecast model for C/D ratios in stationary phase. Recipient SLCO1B1 rs2291075 genotype had significant effect on tacrolimus C/D ratios in convalescence phase (*p* = 0.0300) and stationary phase (*p* = 0.0400) in subgroup, which excluded the interference come from donor and recipient CYP3A5 rs776746.

**Conclusion:**

SLCO1B1 rs2291075 could be a novel genetic locus associated with tacrolimus metabolism. The combined analysis of donor and recipient CYP3A5 rs776746, recipient SLCO1B1 rs2291075 genotypes, could be helpful to guide the personalized administration of tacrolimus in early period after liver transplantation.

**Supplementary Information:**

The online version contains supplementary material available at 10.1007/s00228-020-03058-w.

## Introduction

Liver transplantation is the method of choice in the treatment of patients with end-stage liver failure and selected group of patients with hepatocellular carcinoma (HCC) [1, 2]. Tacrolimus is a calcineurin inhibitor and the main immunosuppressant drug used after liver transplantation. Adequate immunosuppression is necessary to prevent rejection and to increase the survival rate of transplantation; overimmunosuppression could give rise to a series of serious adverse drug reactions, such as infection, diabetes, and renal insufficiency. However, the narrow therapeutic index and large interindividual variabilities of tacrolimus complicate its routine dosage adjustment [3–6].

Therapeutic drug monitoring (TDM) is the most common strategy for immunosuppressive therapy in daily clinical practice and effectively ameliorates drug efficacy and safety [7, 8]. At one time, the dosage of tacrolimus was adjusted continuously guided by monitoring trough blood concentrations in the early period after liver transplantation, and then, the individualized dosage was found. After this period, the clinical value of pharmacogenomics decreased, and the frequency of detection of trough blood concentrations of tacrolimus was reduced. However, this “trial and error” process was prone to result in rejection or adverse drug reactions. Thus, predicting the tacrolimus clearance rate and providing a prospective dosage within an accurate reference range to achieve immune balance in a short time was crucial in immunosuppressive therapy.

In recent years, pharmacogenomics research had provided an active strategy that can forecast drug metabolism phenotypes according to genotype [9, 10]. Enzymes in the cytochrome P450 (CYP) 3A family are responsible for the oxidative metabolism of tacrolimus [11]. Recent guidelines for the use of tacrolimus proposed by the Clinical Pharmacogenetics Implementation Consortium (CPIC) were trying to provide information relevant to the interpretation of CYP3A5 genotype and dosing [12]. The recipients’ metabolism phenotypes were accordingly assigned to 3 categories, including extensive metabolizer (EM, an individual carrying two functional alleles) with CYP3A5 rs776746 AA, intermediate metabolizer (IM, an individual carrying one functional allele and one nonfunctional allele) with CYP3A5 rs776746 AG, and poor metabolizer (PM, an individual carrying two nonfunctional alleles) with CYP3A5 rs776746 GG. This CIPC-EIP classification for tacrolimus is commonly recommended after many organ transplant operations, including kidney, heart, lung, and hematopoietic stem cell transplants, and liver transplants in which the donor and recipient genotypes are identical. However, almost all clinical liver transplantation is allogeneic, and the genotypes of the recipients and their corresponding donors were different. The integration of donor and recipient CYP3A5 rs776746 genotypes could be a more appropriate approach for forecasting tacrolimus metabolism [13, 14].

In our previous study, 115 donors and recipient liver samples in our hospital were genotyped using the Affymetrix DMET Plus microarray (including 1936 genetic variants over 225 related genes) for investigating broad coverage of pharmacogenomic markers and screening unknown genes may be involved in tacrolimus metabolism. Tacrolimus trough blood concentration by the corresponding weight-adjusted dosage (C/D ratio) was used as an indicator of drug elimination [15]. Tacrolimus C/D ratios of recipient solute carrier organic anion transporter family member 1B1 (SLCO1B1) rs2291075 CT and TT carriers were very close and were significantly lower than those of CC genotype carriers in week1 and week 2 post-operation (*p* = 0.0349, and *p* = 0.0041, respectively). There were no statistically significant differences between tacrolimus C/D ratios of donors with SLCO1B1 rs2291075 CT, TT genotype, and those with CC genotype carriers in week 1 and week 2 post-operation.

The SLCO1B1 gene which is located on chromosome 12p12.1 encodes the protein of organic anion transporting polypeptide 1B1 (OATP1B1), an influx transmembrane protein transporter. Several groups of the influx transporters uptake a variety of drugs and organic compounds from the blood into the cell, especially the organic anion transporting polypeptides (OATPs) which are expressed in many organs such as the intestine, liver, and kidneys. OATPs have shown an important role in clinical implications for the pharmacokinetics of many drugs, including drug absorption, distribution, and elimination. Evidence of changes in OATP transport function has been found to affect the efficacy and safety of many drugs and leading to instability in drug disposition and response. SLCO1B1 was expressed in both the liver and intestine [16–18]. SLCO1B1 rs2291075 was a functional genetic polymorphism associated with treatment outcome in acute myeloid leukemia and urine arsenic metabolites [19, 20]. Influence of SLCO1B1 rs2291075 on tacrolimus pharmacokinetic parameters of tacrolimus in liver transplantation was unclear.

In this study, we examined the CYP3A5 rs776746 and SLCO1B1 rs2291075 genotypes of both recipients and donors to clarify the influence of these genetic variants on tacrolimus dose requirements and pharmacokinetics in the early period after liver transplantation.

## Materials and methods

### Patients

210 patients who received orthotopic liver transplantations between January 2015 and December 2019 in Shanghai General Hospital Affiliated to Shanghai Jiao Tong University (*n* = 115) and First Affiliated Hospital of Zhengzhou University (*n* = 95) were enrolled in this retrospective study (148 males and 62 females; mean ± SD age: 48.2 ± 9.5 years; weight: 66.8 ± 11.2 kg). The donor liver and recipient liver tissues were deposited − 80 °C low temperature refrigerator. All patients received tacrolimus, mycophenolate mofetil, and steroids administered at a standard dosage regimen. The initial oral daily dose of tacrolimus was 0.05~0.10 mg/(kg/day) on the first postoperative day. Tacrolimus (Prograf, Astellas Pharma, Tokyo, Japan) was orally administered twice daily. The dosage was adjusted to achieve the target trough level, which was set at between 7 and 10 ng/ml during the first month. Clinical parameters including daily drug dose, serum albumin concentration, hemoglobin, glutamate pyruvate transaminase, and total bilirubin were also recorded at 1–28 days after operation in this study.

### Ethics statement

Liver grafts were obtained from donation after cardiac death (DCD). No donor livers were harvested from executed prisoners. The participants or the next of kin gave their informed consent for the study. The research was approved by the Ethics Committee of Shanghai General Hospital Affiliated to Shanghai Jiao Tong University, the First Affiliated Hospital of Zhengzhou University. The methods were carried out in accordance with the Declaration of Helsinki and its later amendments or comparable ethical standards. This study had register on ClinicalTrials.gov (Identifier: NCT02752529).

### Tacrolimus concentration determinations

The drug trough blood concentration was measured in whole blood in laboratories at each participating center by the Pro-TracTMII tacrolimus ELISA kit (Diasorin, Stillwater, MN, USA) with a microparticle enzyme immunoassay (ELx 800NB analyzer, BioTek, Winooski, VT, USA). The tacrolimus C/D ratio (ng ml^−1^ mg^−1^ kg^−1^) was used as an index of tacrolimus pharmacokinetics and was calculated by dividing the tacrolimus trough blood concentration (ng/ml) by the corresponding weight-adjusted total daily dosage (mg/kg body weight) [21, 22].

### Genotyping

Both donor and recipient genomic DNA were extracted from liver tissues using an AllPrepDNA/RNA Mini Kit (Qiagen, Hilden, Germany). PCR amplification was done for the CYP3A5 and SLCO1B1 genes containing the polymorphisms. Primers were designed as follows. CYP3A5 rs776746: forward 5′-TCTCCCCTCAAGTCCTCAG-3′ and reverse 5′-CCATACCCCTAGTTGTACGAC-3′; SLCO1B1 rs2291075: forward 5′-GTTCATGGGTAATATGCTTCGTGGAATA-3′ and reverse 5′-GCCAAGAATGCATGGTTCTTATTCAC-3′.

The PCR mixture contained 250 ng of genomic DNA. Final volume of 50 μl in reaction buffer contained 5 μl of 10 × PCR buffer, 5 μl of MgCl2 (1 mmol/l), 1 μl of dNTP (10 mM), 2 μl of each primer (10 μm/ml), and 3 U of Taq gold DNA polymerase. Cycling used a GeneAmp PCR system (Applied Biosystems, Waltham, MA, USA) as follows: initial denaturation at 94 °C for 3 min; 35 cycles at 94 °C for 30 s, 55 °C for 30 s, 72 °C for 1 min; and a final step at 72 °C for 10 min. The amplified PCR products were sequenced using an ABI 3730-XL Automated Sequencer (Applied Biosystems, Waltham, MA, USA).

### Statistical analysis

The early postoperative period after liver transplantation was divided into convalescence phase (1–14 days) and stationary phase (15–28 days) according to the change of liver function and tacrolimus C/D values [6]. A chi-squared test was used to compare the distributions of genotypes and alleles of recipients and donors. A Mann–Whitney *U* test was used to investigate the difference between the two groups, and a Kruskal–Wallis test was used to assess the statistical significance among several groups. Multivariate analysis was conducted using the logistic regression model. Statistical analysis was performed using SPSS version 17.0 (SPSS Inc., IL, USA). Hardy–Weinberg equilibrium, allele frequency, linkage disequilibrium was analyzed using SHEsis software (http://analysis.bio-x.cn). *p* < 0.05 was considered statistically significant. The statistical graphs were made by Graphpad Prism version 5.00 software (GraphPad Software Inc., San Diego, CA, USA).

## Results

### Frequencies of CYP3A5 and SLCO1B1 polymorphisms

The distributions of CYP3A5 rs776746 and SLCO1B1 rs2291075 genotypes and alleles of recipients and donors are shown in Supplementary Table 1. There were no significant deviations from Hardy–Weinberg equilibrium for any of the allelic frequencies for recipients and donors (*p* > 0.05). No statistically significant differences in genotype and allele frequencies were observed between recipients and donors for any of CYP3A5 rs776746 and SLCO1B1 rs2291075 polymorphisms.

### Effects of single polymorphism on tacrolimus C/D ratios

**Fig. 1:**
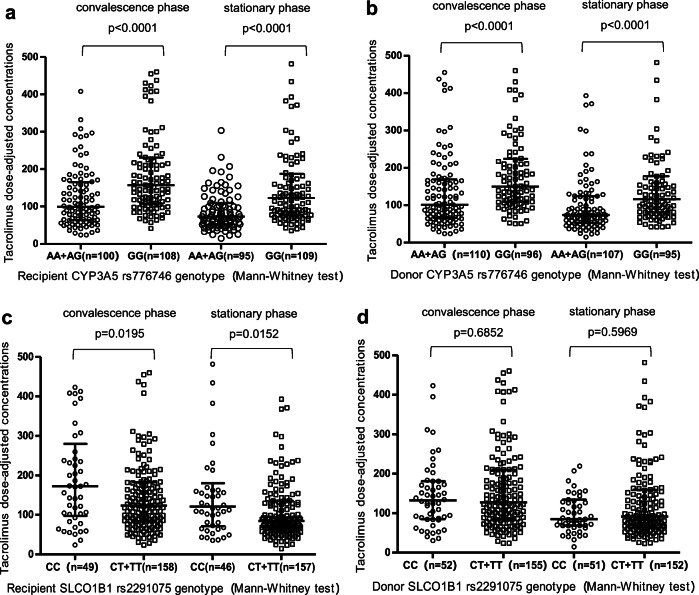
Influence of donor and recipient CYP3A5 rs776746 and SLCO1B1 rs2291075genotype on tacrolimus C/D ratios. **a** Influence of recipient CYP3A5 rs776746 genotype on tacrolimus C/D ratios. **b** Influence of donor CYP3A5 rs776746 genotype on tacrolimus C/D ratios. **c** Influence of recipient SLCO1B1 rs2291075 genotype on tacrolimus C/D ratios. **d** Influence of donor SLCO1B1 rs2291075 genotype on tacrolimus C/D ratios

The effects of single polymorphism on tacrolimus C/D ratios are shown in Fig. 1. Tacrolimus C/D ratios of recipient CYP3A5 rs776746 GG genotype carriers were 157.2 ± 122.7 and 122.6 ± 110.1 in convalescence period and stabilizing period, respectively. Tacrolimus C/D ratios of recipient CYP3A5 rs776746 AA+AG genotype carriers were 99.53 ± 101.3 and 73.00 ± 56.74 in convalescence period and stabilizing period, respectively. The differences were significant at every time point (*p* < 0.0001) (Fig. 1a). Tacrolimus C/D ratios of donors with CYP3A5 rs776746 GG genotype were 149.6 ± 115.7 and 116.2 ± 96.90 in convalescence period and stabilizing period, respectively. For AG and AA genotype carriers, the corresponding tacrolimus C/D ratios at each time point were 101.5 ± 102.8 and 74.20 ± 71.40. The differences were significant (*p* < 0.0001) (Fig. 1b).

Tacrolimus C/D ratios of recipient SLCO1B1 rs2291075 CC, CT, and TT genotype carriers were 172.8 ± 183.2, 118.4 ± 106.3, and 125.8 ± 88.70 in convalescence period and 121.0 ± 108.5, 84.00 ± 70.84, and 88.65 ± 75.08 in stabilizing period, respectively. The differences were borderline significant in convalescence period (*p* = 0.0501) and there were statistically significant differences in stabilizing period (*p* = 0.0318). Tacrolimus C/D ratios of recipient SLCO1B1 rs2291075 CT and TT carriers were very close and were significantly lower than those of CC genotype carriers in convalescence period (*p* = 0.0195) and in stabilizing period (*p* = 0.0152) (Fig. 1c). There were no statistically significant differences between tacrolimus C/D ratios of donors with SLCO1B1 rs2291075 CT, TT genotype, and those with CC genotype carriers (Fig. 1d) at each period (*p* = 0.6852 and 0.5969, respectively).

### Combined effect of donor and recipient CYP3A5 rs776746 polymorphisms on tacrolimus C/D ratios

According to the literature, CYP3A5 rs776746 AA and AG genotype expressed an active CYP3A5 enzyme and GG genotype resulted in a splicing defect and the production of a truncated nonactive protein with no CYP3A5 enzyme activity [12]. All patients were divided into 4 groups according to donor and recipient CYP3A5 rs776746 genotype: recipient and donor expressed active CYP3A5 enzyme (Re e + Do e); recipient or donor expressed active CYP3A5 enzyme (Re e + Do n, Re n + Do e); and neither recipient nor donor expressed active CYP3A5 enzyme (Re n + Do n). There were significant differences among the four groups in convalescence period (*p* < 0.0001, Supplementary Fig. 1a) and stabilizing period (*p* < 0.0001, Supplementary Fig. 1b). There were no significant differences between the Re e + Do n and Re n + Do e group in convalescence period and stabilizing period (*p* = 0.5917 and 0.7164, respectively, Supplementary Fig. 1a, b); the two groups were merged. Then, a FIS genotype classification system associated with tacrolimus elimination phenotype was built: fast elimination (FE) group with low C/D ratios (Re e + Do e genotype), intermediate elimination (IE) group with medium C/D ratios (Re e + Do n or Re n + Do e genotype), and slow elimination (SE) group with high C/D ratios (Re n + Do n genotype). The differences were significant at every period (*p* < 0.0001) (*p* < 0.0001, Supplementary Fig. 1c, d).

### Multivariate analysis

Multivariate linear regression analysis was used to screen the influence factors of tacrolimus pharmacokinetics. The incorporated cofactors included daily drug dose, serum albumin concentration, hemoglobin, glutamate pyruvate transaminase, and total bilirubin; all reported to have the potential effects on tacrolimus pharmacokinetics [6, 8], as well as FIS genotype classification and recipient SLCO1B1 rs2291075. The results (Table 1) have shown that daily dose, FIS genotype classification, and total bilirubin were the consistent predictors for tacrolimus elimination in convalescence period and stabilizing period after liver transplantation. Recipient SLCO1B1 rs2291075 was confirmed to be an independent predictor of C/D ratios in convalescence period.

**Table 1:** Prediction model of tacrolimus elimination in the early postoperative period after liver transplantation

		Unstandardized coefficients		Standardized coefficients	*t*	*p*
		*B*	Std. error	Beta		
Convalescence period	Constant	2.259	0.085		26.594	0.000
	Daily dose	− 5.806	0.546	− 0.535	− 10.636	0.000
	FIS classification	0.112	0.021	0.266	5.435	0.000
	Total bilirubin	0.001	0.000	0.247	5.303	0.000
	SLCO1B1 rs2291075	− 0.075	0.032	− 0.105	− 2.319	0.021
Stationary period	Constant	2.103	0.069		30.392	0.000
	Daily dose	− 5.436	0.460	− 0.598	− 11.819	0.000
	FIS classification	0.118	0.023	0.261	5.198	0.000
	Total bilirubin	0.001	0.000	0.189	4.013	0.000

### Slow elimination subgroup multivariate analysis

Analysis indicators included daily drug dose, serum albumin concentration, hemoglobin, glutamate pyruvate transaminase, total bilirubin, and recipient SLCO1B1 rs2291075. This slow elimination (SE) subgroup multivariate analysis explored the influence of recipient SLCO1B1 rs2291075 genotype on tacrolimus C/D ratios excluded the interference that comes from donor and recipient CYP3A5 rs776746. Variables of SLCO1B1 rs2291075, total bilirubin, and daily dose constituted prediction model of tacrolimus elimination in SE subgroup. Recipient SLCO1B1 rs2291075 was an independent predictor of C/D ratios of SE subgroup in convalescence period (*p* = 0.030) and stabilizing period (*p* = 0.040) (Table 2).

**Table 2:** Prediction model of tacrolimus elimination in slow elimination (SE) subgroup

		Unstandardized coefficients		Standardized coefficients	*t*	*p*
		*B*	Std. error	Beta		
Convalescence phase	Constant	2.749	0.113		24.260	0.000
	SLCO1B1 rs2291075	− 0.123	0.055	− 0.231	− 2.251	0.030
	Total bilirubin	0.001	0.000	0.366	3.561	0.001
	Daily dose	− 8.686	1.517	− 0.588	− 5.727	0.000
Stationary phase	Constant	2.728	0.122		22.440	0.000
	SLCO1B1 rs2291075	− 0.138	0.065	− 0.226	− 2.119	0.040
	Total bilirubin	0.002	0.001	0.256	2.458	0.018
	Daily dose	− 7.007	1.278	− 0.583	− 5.483	0.000

## Discussion

In this study, we demonstrated the influence of recipient SLCO1B1 rs2291075 polymorphisms on tacrolimus C/D ratios based on pieces of evidence from the three levels of analysis: Firstly, recipient SLCO1B1 rs2291075 polymorphisms were associated with tacrolimus C/D ratios by univariate correlation analysis. Secondly, SLCO1B1 rs2291075 polymorphisms were an independent factor that might predict tacrolimus C/D ratios by multivariate linear regression analysis included known influencing factors (daily drug dose, serum albumin concentration, hemoglobin, glutamate pyruvate transaminase, total bilirubin, and CYP3A5 rs776746 genotype). Thirdly, the influence of recipient SLCO1B1 rs2291075 genotype on tacrolimus C/D ratios was confirmed in the slow elimination subgroup, which excluded the interference come from donor and recipient CYP3A5 rs776746. Two studies of renal transplant patients illustrated that SLCO1B1 polymorphisms were associated with tacrolimus concentrations, and genotyping for SLCO1B1 SNPs may be useful for individualized medicine of tacrolimus [23, 24]. There are obvious differences in influence factors of tacrolimus elimination between liver transplantation and renal transplantation due to the state of metabolic organ (the liver and intestine), internal environment, and dose required. To our knowledge, this is the first study of the association between SLCO1B1 polymorphisms and tacrolimus elimination in liver transplantation.

During recent years, increasing pieces of evidence showed that drug transporters played important roles in absorption and distribution of multiple drugs. Organic anion transporting polypeptides (OATPs) are central membrane transporters involved in the cellular uptake of drugs in tissues (such as the liver, intestine, and kidneys), which is important for pharmacokinetics. SLCO1B1 gene produces the protein of organic anion transporting polypeptide 1B1 (OATP1B1) and is involved in various kinds of drug disposition [25, 26]. SLCO1B1 polymorphisms were a valuable pharmacogenetic marker of statin drugs, a kind of lipid-lowering drug: The SLCO1B1 c.1929C allele was associated with a 75% reduction in rosuvastatin exposure when compared to individuals carrying the wild type SLCO1B1 c.1929A allele [27]. There was a significant association between the SLCO1B1 c.521T>C polymorphism and atorvastatin-related adverse drug reactions (ADRs) associated with risk allele. Allele C was associated with increased lipid-lowering efficacy in people with hyperlipidemias as compared to allele T [28]. R4149056T>C in SLCO1B1 increased systemic exposure to simvastatin and the risk of muscle toxicity. The SLCO1B1 c.521T>C variant significantly increased exposure to simvastatin acid by around 40% [29, 30]. These results suggested that genotyping SLCO1B1 polymorphisms were essential for individual statin therapy. The relationship between SLCO1B1 polymorphisms and drug hepatotoxicity was also observed. SLCO1B1 rs4149056 genetic polymorphism could predict methotrexate hepatotoxicity in Chinese patients with non-Hodgkin lymphoma. Patients with TC and CC genotype had more hepatotoxicity than TT genotype (60% vs 32.94%) [31]. Rs2306283 of SLCO1B1 exhibited a significant association with methimazole-induced liver injury [32]. Through the above research data, it could be drawn that the polymorphisms of SLCO1B1 gene were associated with drug safety and efficacy.

According to the report by Shu Liu et al., SLCO1B1 rs2306283 polymorphisms were associated with tacrolimus concentrations in Chinese renal transplant recipients [23]. In the present study, we firstly found the effect of recipient SLCO1B1 rs2291075 polymorphisms on tacrolimus elimination in Chinese liver transplant patients.

Rs2291075, a genetic variant in SLCO1B1, has been reported as a tag SNP to distinguish the three major SLCO1B1 haplotypes (*1A, *1B, and *15). Additionally, rs2291075, among several other variants in the SLCO1B3-SLCO1B1 genomic region, is significantly associated with SLCO1B1 protein expression. The transporter SLCO1B1 has an important role in the system pharmacokinetics of multiple drugs used in the treatment of acute myeloid leukemia (AML). AML patients harboring two copies of the rs2291075 variant (CC) had a significantly more favorable survival outcome compared to individuals who are heterozygous (CT) or noncarriers (TT) [19]. In another study, the association between SLCO1B1 rs2291075 and arsenic metabolites was observed [20]. Therefore, SLCO1B1 rs2291075 could be functional SNP.

Tacrolimus was metabolized in the recipient intestine and donor liver in liver transplantation patients [33]. In most cases, the SLCO1B1 rs2291075 genotypes of recipient intestine and donor liver were different. Tacrolimus C/D ratios of recipient SLCO1B1 rs2291075 CT and TT carriers were significantly lower than those of CC genotype carriers in convalescence period (*p* = 0.0195) and in stabilizing period (*p* = 0.0152). However, there were no statistically significant differences between tacrolimus C/D ratios of donors with SLCO1B1 rs2291075 CT, TT genotype, and those with CC genotype carriers at each period. The expression of SLCO1B1 was often thought to be liver-specific. However, SLCO1B1 mRNA could be expressed in a variety of tissues and cells according to data of GENECARDS (https://www.genecards.org/cgi-in/carddisp.pl?gene=SLCO1B1&keywords=SLCO1B1). A study showed that SLCO1B1 mRNA was expressed in the islets in very low quantities (< 1% relative to the liver control sample). The quantity of SLCO1B1 in the islets was significantly reduced by the presence of sirolimus, and tacrolimus antagonizes the sirolimus inhibition of OATP1B1 expression. SLCO1B1 in the islets might involve in the drug–drug interaction of tacrolimus and sirolimus [34]. The SLCO1B1 mRNA expression was also readily detected in intestinal biopsies using reverse transcriptase–polymerase chain reaction [35]. We inferred that SLCO1B1 could participate in tacrolimus absorption in the intestine. Further study should explore the protein expression of SLCO1B1 and its influence on the process of tacrolimus in the body.

In this study, both donor and recipient CYP3A5 rs776746 genotypes were confirmed influencing tacrolimus elimination in the early period after liver transplantation [36]. Moreover, donor and recipient CYP3A5 rs776746 polymorphisms were combined to build initial FIS classification systems to more effectively distinguish tacrolimus elimination phenotype: fast elimination (FE, recipient of CYP3A5 rs776746 AA/AG with CYP3A5 rs776746 AA/AG donor), intermediate elimination (IE, recipient of CYP3A5 rs776746 GG with CYP3A5 rs776746 AA/AG donor or recipient of CYP3A5 rs776746 AA/AG with CYP3A5 rs776746 GG donor), and slow elimination (SE, recipient of CYP3A5 rs776746 GG with CYP3A5 rs776746 GG donor). This FIS classification system had a higher distinguished ability of tacrolimus dosage than only donor CYP3A5 rs776746 or recipient CYP3A5 rs776746, as is more precise and effective in guiding individualized tacrolimus use. FIS classification was used as genetic factors influencing tacrolimus elimination in multivariate analysis in the present study.

A model consisting of tacrolimus daily dose, total bilirubin, FIS classification, and recipient SLCO1B1 rs2291075 could predict tacrolimus C/D ratios in convalescence phase by multivariate analysis. However, recipient SLCO1B1 rs2291075 genotype failed to enter forecast model for C/D ratios in stationary phase. Recipient SLCO1B1 rs2291075 genotype had significant effect on tacrolimus C/D ratios in convalescence phase and stationary phase in SE subgroup, which excluded the interference come from donor and recipient CYP3A5 rs776746. The CYP3A5 protein expression quantity was reduced obviously in donor liver and recipient intestine in convalescence phase, which resulted in high tacrolimus C/D ratios. And the CYP3A5 protein produced by donor liver and recipient intestine cells returned to nearly normal levels in stationary phase [6]. The quantity of functional CYP3A5 was very low in SE subgroup liver transplantation patients due to donor and recipient CYP3A5 rs776746 polymorphisms which produced in CYP3A5 protein without enzyme activity. Our data suggested that SLCO1B1 rs2291075 had a better ability to predict for tacrolimus C/D ratios in convalescence phase and SE subgroup which tacrolimus had a slow metabolism on account of poor CYP3A5 protein enzyme activity.

Tacrolimus daily dose and total bilirubin were the main clinical influencing factors of tacrolimus C/D ratios in multivariate analysis. This result was consistent with previous studies. Tacrolimus was metabolized by the liver microsomal cytochrome P450 enzyme system and the metabolite of tacrolimus was excreted with bile. High total bilirubin level indicted reducing drug excretion and slow drug clearance rate. There was a positive feedback in tacrolimus metabolism: drug elimination rate accelerated with the increase of dose [8, 11, 37].

There are some limitations to the study: Firstly, microparticle enzyme immunoassay was used to detect tacrolimus metabolites. The cross-reactivity with various tacrolimus metabolites of the antibody might have a potential impact on the testing result. Secondly, the drug–drug interaction (DDI) and indexes of inflammation, which had some effects on tacrolimus pharmacokinetics, should be included in multivariate analysis in further study. Thirdly, the large sample multicenter study was needed to confirm the relationships between recipient SLCO1B1 rs2291075 polymorphism and tacrolimus dose–corrected trough concentration in the early period after liver transplantation.

In conclusion, SLCO1B1 rs2291075 could be a novel genetic locus associated with tacrolimus metabolism. Recipient SLCO1B1 rs2291075 was an important genetic factor under the circumstances of poor CYP3A5 protein enzyme activity (liver transplantation patients in convalescence phase, SE subgroup patients). The combined analysis of donor and recipient CYP3A5 rs776746, recipient SLCO1B1 rs2291075 genotypes, could be helpful to guide the personalized administration of tacrolimus in the early period after liver transplantation.

## Supplementary Information

ESM 1 (PDF 101 kb)

ESM 2 (DOCX 189 kb)
