# Supplementary material for: The influence of recipient SLCO1B1 rs2291075 polymorphism on tacrolimus dose–corrected trough concentration in the early period after liver transplantation
Source: Eur J Clin Pharmacol. 2021 Jan 2;77(6):859–67. doi: 10.1007/s00228-020-03058-w (PMC8128732; doi:10.1007/s00228-020-03058-w)
Supplement: Supplementary file 1 — (PDF 101 kb) [file 228_2020_3058_MOESM1_ESM.pdf]

Supplementary table 1 Genotype and allele frequency of SLCO1B1 and CYP3A5 polymorphism in liver transplant recipients (n = 210) and donors (n =210).

SNP	Genotype frequency, %			Allele frequency, %	
Recipient CYP3A5 rs776746	AA(9.5)	AG(38.1)	GG(52.4)	A(28.6)	G(71.4)
Donor CYP3A5 rs776746	AA(6.3)	AG(46.6)	GG(47.1)	A(29.6)	G(70.4)
Recipient SLCO1B1 rs2291075	CC(23.9)	CT(54.6)	TT(21.5)	C(51.2)	T(48.8)
Donor SLCO1B1 rs2291075	CC(25.4)	CT(49.2)	TT(25.4)	C(50.0)	T(50.0)
