# Supplementary material for: The influence of recipient SLCO1B1 rs2291075 polymorphism on tacrolimus dose–corrected trough concentration in the early period after liver transplantation
Source: Eur J Clin Pharmacol. 2021 Jan 2;77(6):859–67. doi: 10.1007/s00228-020-03058-w (PMC8128732; doi:10.1007/s00228-020-03058-w)
Supplement: Supplementary file 2 — (DOCX 189 kb) [file 228_2020_3058_MOESM2_ESM.docx]

b

a

p<0.0001 (Kruskal-Wallis test)

p=0.5917 (Mann Whitney test)

p<0.0001 (Kruskal-Wallis test)

p=0.7164 (Mann Whitney test)

p<0.0001 (Mann Whitney test)

p<0.0001 (Kruskal-Wallis test)

p<0.0001 (Mann Whitney test)

p<0.0001 (Kruskal-Wallis test)

p<0.0001 (Mann Whitney test)

p<0.0001 (Mann Whitney test)

d

c

Supplementary Fig.1 Combined effect of donor and recipient CYP3A5 rs776746 polymorphisms on tacrolimus C/D ratios

a and b: Combined effect of donor and recipient CYP3A5 rs776746 polymorphisms on C/D ratios in convalescence phase (c) and stationary phase (d) (Re e: recipient CYP3A5 rs776746 AA+AG; Re n: recipient CYP3A5 rs776746 GG; Do e: Donor CYP3A5 rs776746 AA+AG; Do n: Donor CYP3A5 rs776746 GG)

c and d: FIS classification systems were built in convalescence phase (e) and stationary phase (f) (FE: Re e + Do e, IE: Re e + Do n or Re n + Do e, SE: Re n+ Do n)
